# Penetrating pulmonary injury caused by a steel rod

**DOI:** 10.1002/rcr2.640

**Published:** 2020-08-18

**Authors:** Dario Amore, Emanuele Muto, Dino Casazza, Marcellino Cicalese, Marco Rispoli, Carlo Curcio

**Affiliations:** ^1^ Department of Thoracic Surgery Monaldi Hospital Naples Italy; ^2^ Department of Diagnostic Imaging, General Radiology Monaldi Hospital Naples Italy; ^3^ Department of Anesthesia and Intensive Care Monaldi Hospital Naples Italy

**Keywords:** Lung injury, radiology and other imaging, thoracic surgery

## Abstract

In stable patients with penetrating thoracic trauma, a careful radiological assessment should be taken into account for a correct surgical management.

## Graphical abstract

Penetrating thoracic injuries by metallic rod are relatively rare. Here, we present the case of a stable patient with penetrating thoracic trauma where a careful radiological assessment helped us to perform a correct surgical management.

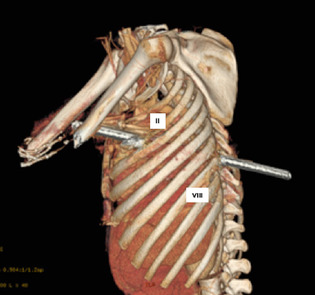

## Clinical Image

**Figure 1:**
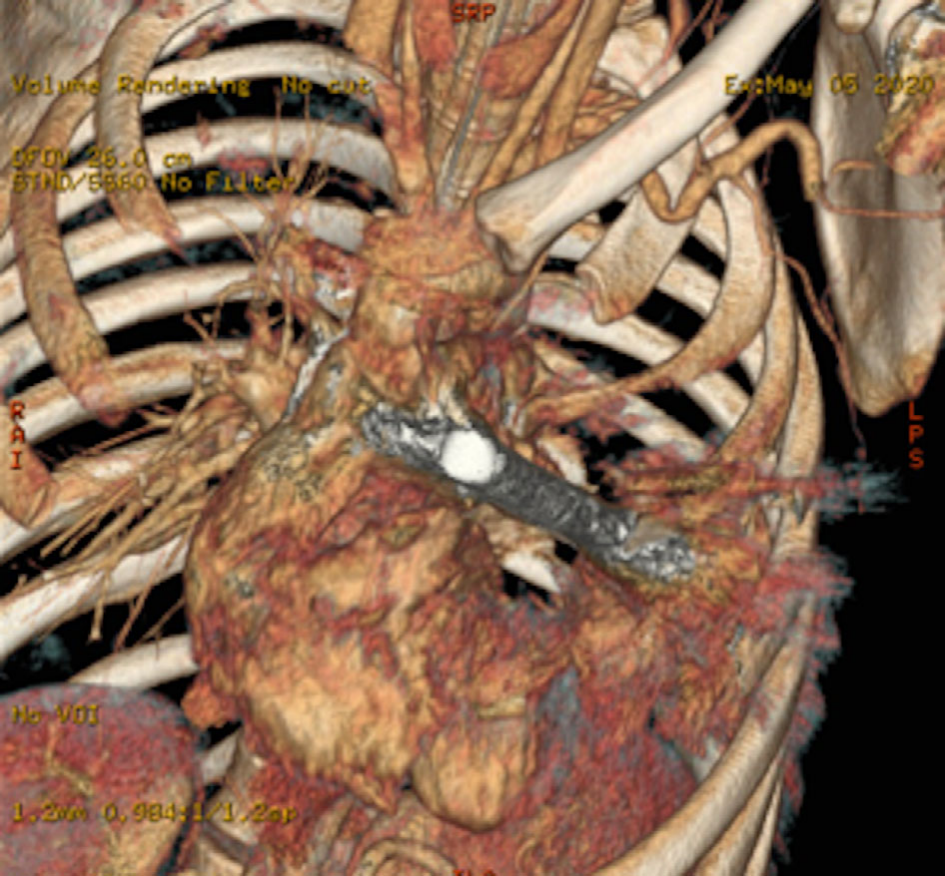
Three‐dimensional (3D) volume rendering of chest computed tomography (CT) scan shows the relation of the foreign body with heart and the metal‐related artefacts.

**Figure 2:**
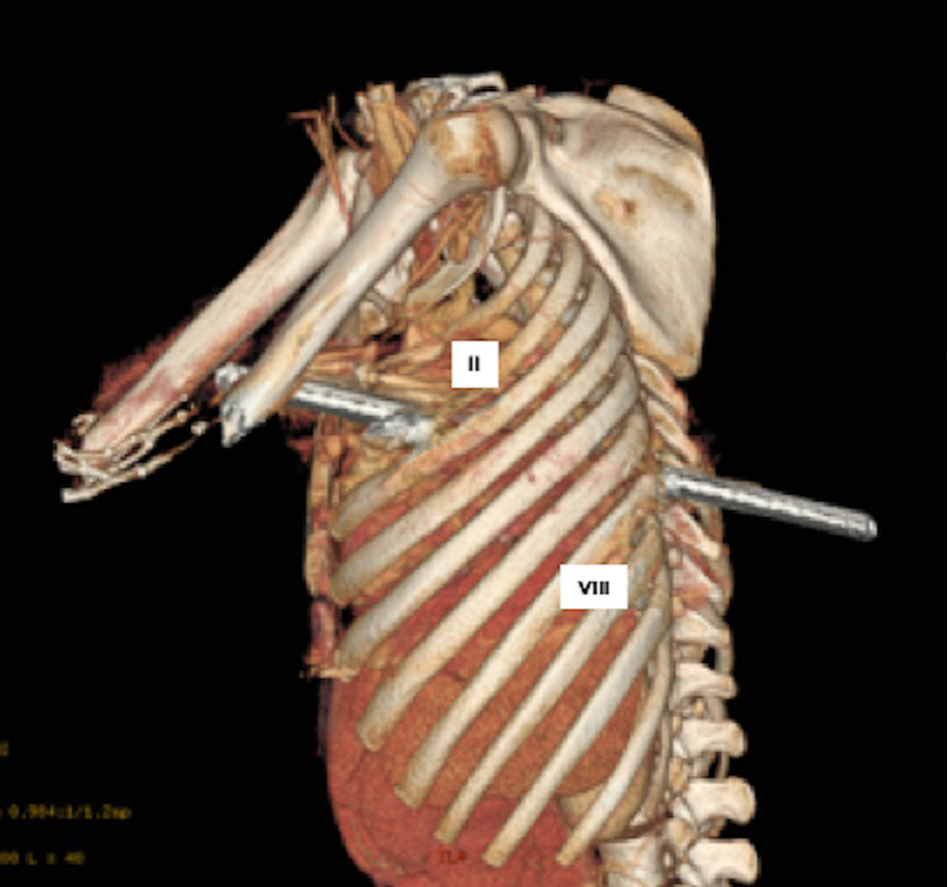
Three‐dimensional (3D) visualization of computed tomography (CT) scan reveals a rod penetrating left hemithorax through the eighth intercostal space of the posterior chest wall and coming out from the second intercostal space of the anterior chest wall. II, second intercostal space; VIII, eighth intercostal space.

**Figure 3:**
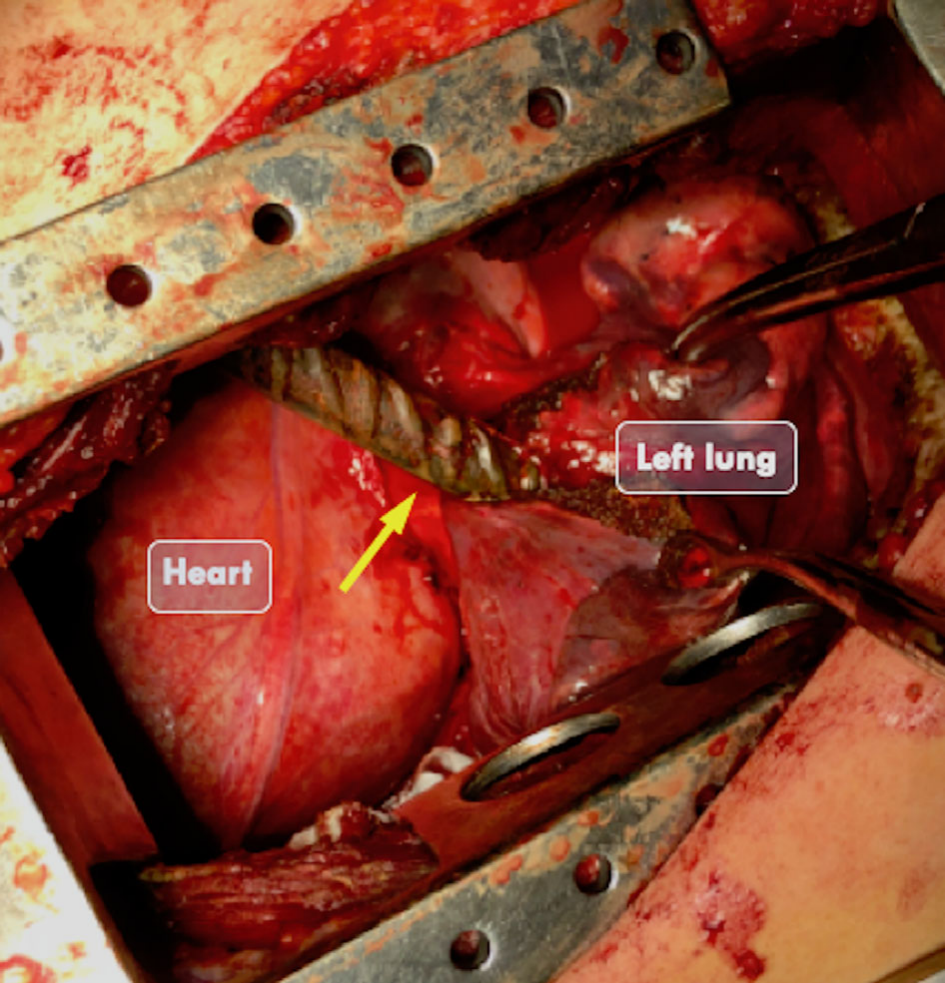
Intraoperative view after left thoracotomy. The steel rod (yellow arrow) passes through the pleural cavity penetrating the lung and without involving the heart.

**Figure 4:**
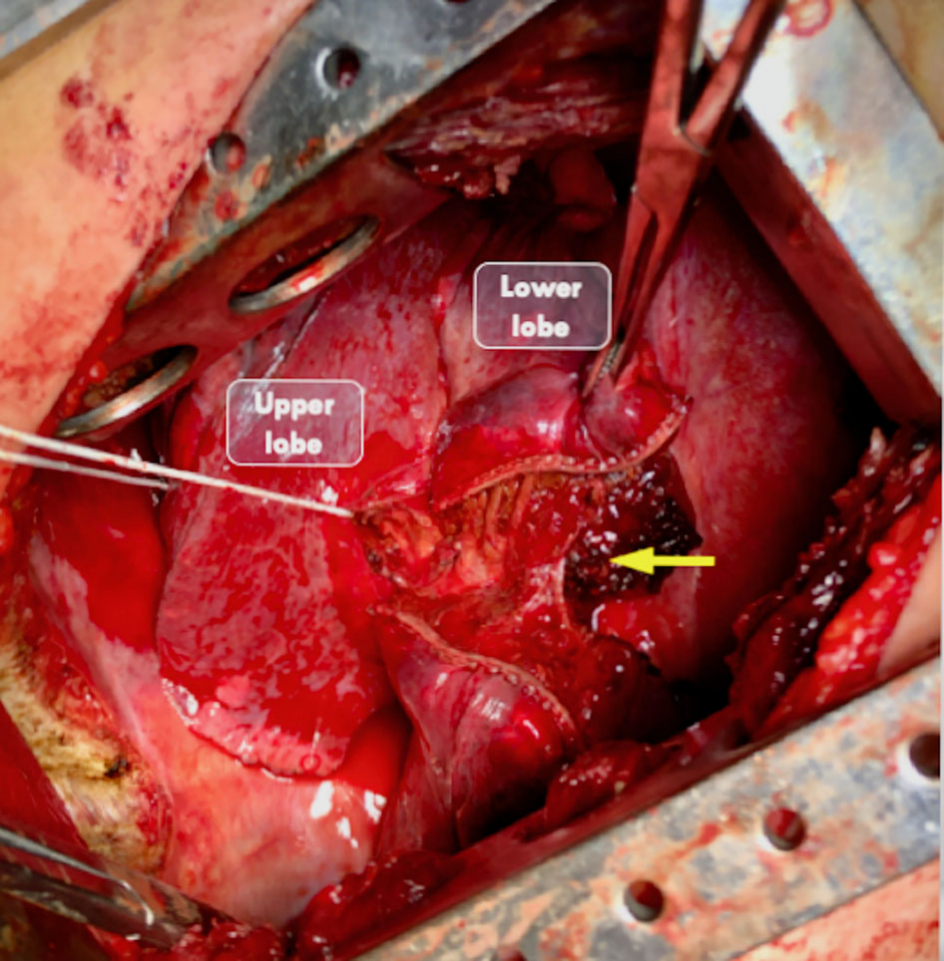
Intraoperative photograph after bar removal. The tract of left lower lobe traversed by the rod is opened with a stapling device (yellow arrow).

A 22‐year‐old construction worker slipped off a scaffold and, falling backwards from a height of 3 m, had a penetrating thoracic injury caused by a steel rod projecting out of a pillar. He was taken to our hospital by ambulance with the metallic object left in situ. On admission, he was fully conscious and haemodynamically stable. After induction of general anaesthesia, a double‐lumen tube was inserted with the patient in the lateral position. Computed tomography (CT) angiography excluded extrathoracic, cardiovascular and neurovascular injuries, showing a metallic foreign body in the chest (Figs 1, 2). The patient was shifted to the operating room and an early thoracotomy was performed: the steel bar, passing through the left lower and upper lobes (Fig. 3), was removed under direct vision and a pulmonary tractotomy was then performed, dividing the lung between the entrance and exit wounds with a stapler (Fig. 4). This technique allowed to open the tract of lung traversed by the rod and to carry out a selective ligation of injured vessels and bronchi, sparing lung tissue. Penetrating thoracic injuries by metallic rod are relatively rare [1, 2]. In stable patient, an assessment of the trauma‐induced injuries may help to plan an appropriate surgical approach.

### Disclosure Statement

Appropriate written informed consent was obtained for publication of this case report and accompanying images.
